# Defective antifungal immunity in patients with COVID-19

**DOI:** 10.3389/fimmu.2022.1080822

**Published:** 2022-11-30

**Authors:** Charles Oliver Morton, James S. Griffiths, Juergen Loeffler, Selinda Orr, P. Lewis White

**Affiliations:** ^1^ Western Sydney University, School of Science, Campbelltown, NSW, Australia; ^2^ Centre for Host-Microbiome Interactions, Faculty of Dentistry, Oral and Craniofacial Sciences, London, United Kingdom; ^3^ Department of Internal Medicine II, University Hospital of Würzburg, Würzburg, Germany; ^4^ Wellcome-Wolfson Institute for Experimental Medicine, School of Medicine, Dentistry and Biomedical Science, Queen’s University Belfast, Belfast, United Kingdom; ^5^ Public Health Wales, Microbiology Cardiff, Wales, United Kingdom

**Keywords:** invasive fungal disease, COVID-19, immune exhaustion, aspergillosis, mucormycosis, antifungal immunity, candidiasis

## Abstract

The COVID-19 pandemic has placed a huge strain on global healthcare and been a significant cause of increased morbidity and mortality, particularly in at-risk populations. This disease attacks the respiratory systems and causes significant immune dysregulation in affected patients creating a perfect opportunity for the development of invasive fungal disease (IFD). COVID-19 infection can instill a significant, poorly regulated pro-inflammatory response. Clinically induced immunosuppression or pro-inflammatory damage to mucosa facilitate the development of IFD and *Aspergillus*, Mucorales, and *Candida* infections have been regularly reported throughout the COVID-19 pandemic. Corticosteroids and immune modulators are used in the treatment of COVID-19. Corticosteroid use is also a risk factor for IFD, but not the only reason for IFD in COVID -19 patients. Specific dysregulation of the immune system through functional exhaustion of Natural killer (NK) cells and T cells has been observed in COVID-19 through the expression of the exhaustion markers NK-G2A and PD-1. Reduced fungicidal activity of neutrophils from COVID-19 patients indicates that immune dysfunction/imbalance are important risk factors for IFD. The COVID-19 pandemic has significantly increased the at-risk population for IFD. Even if the incidence of IFD is relatively low, the size of this new at-risk population will result in a substantial increase in the overall, annual number of IFD cases. It is important to understand how and why certain patients with COVID-19 developed increased susceptibility to IFD, as this will improve our understanding of risk of IFD in the face of future pandemics but also in a clinical era of increased clinical immuno-suppression/modulation.

## 1 Introduction

In the century between the Spanish influenza pandemic and the current coronavirus disease 2019 (COVID-19) pandemic the knowledge of infectious diseases has grown immensely (1). The advent of antibiotic drugs massively reduced the burden of infectious diseases in the developed world and vaccinations campaigns as epitomized by on-going COVID-19 mass immunization campaigns have helped control and even eradicate certain infections (2). Effective treatment for infectious disease has significantly enhanced health care, making treatments such as solid-organ and stem-cell transplants possible, but the increasing population of immunosuppressed or immune dysregulated patients have led to a rise in severe human fungal infections (3). It appears that many medical advances are associated with fungal disease, with a rise in fungal disease also echoed in plants and animals over the same period (4).

While the primary risk-factors for the development of invasive fungal disease (IFD) are clinical interventions causing anatomical disruption or immunosuppression/immune dysregulation of the individual (e.g. neutropenia, graft versus host disease, corticosteroids, monoclonal antibodies) (5–8), in uncontrolled conditions (e.g. Type 2 Diabetes mellitus) there is a risk of IFD (e.g. mucormycosis) due to immune dysfunction in this population (9). The primary AIDS defining diseases remain fungal (e.g. *Pneumocystis* pneumonia and Cryptococcosis) and other viral infections have also been associated with IFD in immunocompromised patients (e.g. cytomegalovirus (CMV) and respiratory viruses in haematology patients). More recently, invasive aspergillosis (IA) has been significantly associated with influenza infection in the intensive care patient and there has been a large number of publications describing IFD in the COVID-19 patient. In these cohorts, it is important to understand why these opportunistic IFD occurred in patients lacking the typical risk factors for IFD. Determining whether antifungal immunity has been compromised by clinical intervention or by the virus is essential for identifying patients at risk of IFD but also in preparation for future viral pandemics. This review will summarize the current knowledge on viral/fungal coinfections before focusing on the impact of COVID-19 infection and its clinical management on antifungal immunity.

### 1.1 Viral infection as a risk factor for IFD

Viral infection has been associated with fungal co-infection for decades. The activity of viral infections can lead to significant disruption of normal immune function and cause damage to epithelial surfaces. These create conditions favorable for fungal infection, although usually in patients with specific underlying conditions (CMV or respiratory virus infection and aspergillosis in the haematology cohort). These co-infecting fungi are frequently described as causing superinfections with negative effects on patient outcomes.

In the AIDS pandemic, fungal infection was a prominent indicator of late stage HIV infection, with *Candida albicans* presenting as a prominent superficial co-infection in HIV positive individuals and systemic disease due to *Pneumocystis* pneumonia and cryptococcal meningitis were, and remain, major AIDS defining diseases (10, 11). The immune disruption resulting from the low CD4 count and the effect of HIV infection on both myeloid and lymphoid immunity impacts both innate and adaptive antifungal immunity with cytokine production, T-Cell and B-Cell responses all affected. It is a major risk factor for fungal infections, which is particularly concerning with antifungal resistance increasing, (e.g. *Candida* spp. such as *C. glabrata* and *C. parapsilosis* have emerged as causes of candidiasis in this cohort) (10, 11). Other fungi endemic to certain geographical areas (e.g. *Histoplasma capsulatum*, and *Talaromyces marneffei*) also cause systemic infections in the HIV population and are associated with significant morbidity and mortality in developing countries (10, 12), although the burden of these mycoses has been reduced in developed countries due to the availability of antiretroviral therapy (13).

CMV infection is a well-documented predisposing factor for IFD in haematology patients post allogeneic stem cell transplantation (SCT), where the function of antigen specific cytotoxic T-Lymphocytes is suppressed along with neutrophil and macrophage responses, and the risk of IFD increased further by neutropenia associated with the use of ganciclovir used to treat the CMV infection (14). In the haematology patient with lymphoproliferative conditions (myeloma/lymphoma) undergoing autologous SCT the risk of both CMV and IFD was considered minimal. However, novel immunomodulatory therapeutic approaches with significant immunosuppressive impact have been associated with increased rates of CMV post autologous SCT and IFD, while still a rare complication in this setting, are associated with CMV reactivation (15). Outside of the haematology population, CMV viraemia has been associated with IA in the critical care patient, where a higher circulating viral load was associated with increased risk of IA (16).

While first described almost 70 years ago, the association between influenza and IA in the critical care setting received significant attention prior to the COVID-19 pandemic that significantly impacted on the incidence of influenza. Influenza associated pulmonary aspergillosis (IAPA) was shown to complicate 19% of patients a median of 3 days post admission to the intensive care unit (ICU) due to influenza and was associated with significant mortality (51%) (17). The use of corticosteroids to suppress the inflammatory reaction to influenza and subsequent symptoms, also suppresses the immune response to fungi and was significantly associated with the development of IAPA. Given corticosteroids are not universally administered in patients with influenza, it cannot be the sole predisposing factor for the development of IAPA. Influenza itself is an independent risk factor for IA and suppresses the nicotinamide adenine dinucleotide phosphate (NADPH) oxidase response to microbial pathogens, and potentially represents a risk factor for IA similar to chronic granulomatous disease (18, 19). Prior to the administration of corticosteroids, the extensive damage to the respiratory epithelium due to influenza/host immune response provides an environment suitable for secondary infection with *Aspergillus*, which has demonstrated the ability to generate large fungal burdens associated with sporulation within the respiratory tract (20). In this context, the administration of corticosteroids would boost infection through immune suppression but possibly also enhancing further fungal growth (21). The use of influenza specific treatments, such as neuraminidase inhibitors (e.g. oseltamivir), have been shown to modulate the host defense against IA by impairing the *Aspergillus* killing capacity of peripheral blood mononuclear cells (22). A recent study investigated the gene expression of 755 genes lined to myeloid innate immunity and performed protein analysis of 47 cytokines, chemokines and growth factors in broncho-alveolar lavage (BAL) fluids from patients with (n=40) and without IAPA (n=52) (23). Patients with IAPA had significantly lower natural killer (NK) cell fractions compared to those from patients with influenza only. There were also numerically lower neutrophil cell fractions and numerically higher epithelial cell fractions in patients with IAPA compared to influenza only. Gene expression analysis, adjusted for the influence of corticosteroids highlighted 26 significantly upregulated and 98 significantly downregulated genes in patients with IAPA, including the downregulation of genes associated with type 1 and type II interferon (IFN) signaling, along with upregulation of fibrosis associated growth factors. Overall, the authors concluded that innate immunity defects in the epithelium, macrophages and neutrophils significantly predisposed patients to IAPA and that recombinant interferon-gamma (IFN-γ) treatment represented a possible adjunct antifungal therapy (23). There is an important role for type I IFNs in immunity to IFD, while viral infection may affect IFN secreting cells there is also a mechanism for reducing IFNs through auto-antibodies. Auto-antibodies produced against cytokines such as the IFNs can effectively disrupt their activity and create another immune dysregulation mechanism that can increase the probability of IFD (24).

### 1.2 COVID-19 and IFD

The COVID-19 pandemic and the sheer scale of infection requiring critical care intervention has created an ideal situation for co-infection by opportunistic pathogens. Secondary fungal infections were documented early during the course of the pandemic, but incidences varied considerably between countries and centres, reflecting different diagnostic approaches, thresholds for positivity and classifications for defining IFD in the COVID-19 patient (25). With cases of COVID-19 associated IFD infrequently supported by autopsy and the lack of antifungal treatment not always associated with a poor prognosis determining an accurate burden of IFD in this cohort is difficult. However, many COVID-19 patients have over-whelming mycological evidence of IFD that would be difficult to ignore even in the absence of autopsy evidence, which may be lacking due to our limited understanding of IFD pathogenesis in this specific cohort (25–27). Given the significant number of post-COVID-19 IFD cases described in the literature and the fact that certain diagnoses are without doubt (e.g. recovery of *Candida* spp. by blood culture), it is safe to say that IFD certainly complicates the management of COVID-19 within critical-care, although it remains difficult to ascertain the actual prevalence.

#### 1.2.1 COVID-19 associated pulmonary aspergillosis

A significant number of studies have described pulmonary aspergillosis secondary to COVID-19 infection, particularly in the critical-care patient, but significant gaps in the knowledge of this condition remain (25). The availability of international definitions for diagnosing CAPA have helped to standardize disease classification and generate a pooled incidence of 7.6% in the critical-care COVID-19 patient, although concerns have been raised regarding the accuracy of CAPA classification according to these definitions, which can define infection based on a single positive mycological criterion, and typical radiological investigations usually providing little clinical utility for identifying CAPA (25, 28–30). CAPA is usually caused by *Aspergillus fumigatus* and presents between 4-11 days after ICU admission, more regularly presenting at the end of the first week/start of the second week (26, 30, 31). Pooled CAPA mortality is 56%, and while a treatment benefit with the recommended aspergillosis antifungal therapies has been demonstrated, not all untreated patients will succumb to infection (25, 29).

#### 1.2.2 COVID-19 associated candidiasis

Both superficial and invasive candidiasis (IC) have been well documented in patients with COVID-19. As with the other IFD, the incidence of CAC varies between centres, but rates are generally higher in critical care settings, likely associated with clinical interventions that increase the risk of IC (25). Given the diagnosis of IC is generally based on the recovery of *Candida* spp. from sterile sites including blood, there should be more confidence in the accuracy of diagnosis. Several studies have confirmed that rates of IC in the ICU during the pandemic were greater than those preceding it but it is not clear if this is directly related to the pathogenesis of COVID-19 itself, or reflects the increased clinical risk factors specific to managing COVID-19 (extracorporeal membrane oxygenation (ECMO), prolonged mechanical ventilation, immunosuppression) or the difficulties experienced by clinical staff managing large patient numbers during the peaks of the pandemic and *Candida* outbreaks have been documented (25, 26, 32–34). The presentation of CAC varies, but median times indicate most cases occur after the first week of ICU admission (26, 32). Mortality varies between 40-70%, and a recent study confirmed that candidaemia during COVID-19 increased all-cause mortality two-fold compared to patients with candidaemia in the absence of COVID-19 (25, 34). *Candida albicans* remains the most prevalent cause of CAC, but rates vary geographical and outbreaks with potentially multidrug resistant *C. auris* have been documented, treatment with an echinocandin remains the frontline option (35, 36).

#### 1.2.3 COVID-19 associated mucormycosis

A substantial number of cases of CAM have been documented in patients with COVID-19 with other underlying conditions that already predispose to mucormycosis, although the estimated incidence of CAM outside of India remains low (<1.0%) (25, 37). CAM usually presents 10-14 days post hospital admission, but can be diagnosed post-COVID-19 recovery (25). COVID-19 patients with poorly controlled diabetes are at particular risk of developing CAM, which usually presents as rhino-orbital cerebral disease, whereas patients with underlying haematological conditions typically present with pulmonary CAM (37). The use of zinc supplements during COVID-19 infection was common in India may also contribute to the development of CAM (38). With diagnosis based on culture, most cases of CAM attain a confidence inspiring proven diagnoses. Most infections are caused by *Rhizopus* spp. All-cause mortality was 49%, ranging from 24% in cases of rhino-orbital CAM, through 59% in cases of rhino-orbital-cerebral CAM, to 81% in cases of pulmonary/disseminated CAM (37). Most cases of CAM are treated with lipid formulations of Amphotericin B, with posaconazole and isavuconazole also used to a lesser extent. However, surgical intervention is recommended and associated with improved outcomes (37).

#### 1.2.4 Other COVID-19 associated fungal infections

While cases of IFD caused by other fungal genera (e.g. *Pneumocystis jirovecii*, *Cryptococcus* spp. *Rhodotorula* spp., *Fusasrium* spp., endemic fungi) have been documented, it is unclear as to whether these infections are directly associated with COVID-19 or simply reflect the huge number of patients infected with COVID-19 across the pandemic and the subsequent increased likelihood of these secondary infections occurring by chance (25). The limited number of cases has prevented further epidemiological, clinical, diagnostic and immunological studies and they will not be considered in detail below.

## 2 Immune dysregulation and risk factors during COVID-19

As highlighted, there has been a considerable number of cases of IFD diagnosed secondary to COVID-19 infection. It is unclear whether this reflects the pathogenesis of COVID-19 creating an environment suited to IFD, if clinical interventions and treatments for COVID-19 increase the risk of IFD or its associated with the presence of pre-existing conditions with established links to IFD within the vast COVID-19 population. On infection, COVID-19 binds to angiotensin-converting enzyme 2 (ACE-2) receptors found on respiratory epithelial cells and leads to transient impairment of ciliary motility, limiting muco-ciliary clearance and innate immune function (39). Cellular uptake and viral replication instigates a pro-inflammatory immune response and in severe cases generates a cytokine storm characterized by increased inflammatory markers including IFN-α, Interleukin (IL)-1Ra and several type 1 (IFN-γ, IL-12p70), type 2 (IL-4, IL-5) and type 3 cytokines (IL-17A, IL-22) and chemokines that direct leukocyte trafficking (C-C Motif Chemokine Ligand 2 (CCL2), C-X-C Motif Chemokine Ligand 9 (CXCL9)) (40). The potential for dysregulation of T-cell response being a risk factor for secondary IFD, is highlighted by consistently elevated IL-17 and IL-22 levels throughout severe COVID-19 infection, compared to a gradual decline in patients with moderate disease (41).

The rapid stimulation of T helper 1 (Th1) cells leads to an imbalanced pro-inflammatory response, with particularly high levels of expression of IL-6 and tumor necrosis factor (TNF), leading to impaired acquired immunity (lymphopenia) and an uncontrolled innate response, further propagated by the release of pro-inflammatory cytokines by alveolar macrophages (25, 42). Dendritic cells may be significantly reduced by COVID-19 infection, with activation of T-cells limited by reduced maturation of, and cytokine release from dendritic cells (43). Defective monocyte and neutrophil function during COVID-19 infection affects monocyte activation and myelopoiesis, leading to a redistribution of the monocyte population and immature circulatory neutrophils and a subsequent hyper-inflammatory response (44). Broncho-alveolar lavage fluid from patients with CAPA can possess significantly lower neutrophil cell fractions and significantly higher epithelial cell fractions compared to patients with only COVID-19 (23). Inflammation increases pulmonary fibrosis and limits repair through enhanced pathological fibroblasts, with prolonged levels of IFN-γ also limiting the repair response (45). Elevated granulocyte macrophage colony-stimulating factor (GM-CSF) has been noted in severe/fatal COVID-19 infection, possibly linked to excessive tissue destruction through elevated monocyte and neutrophil recruitment (46).

The significant damage to the lung parenchyma, exposing the substratum, possibly worsened by the additional inflammatory immune response to the presence of fungal pathogens but also inflammatory fungal toxins (e.g. Mucoricin, gliotoxin), could provide the opportunity for fungi to become pathogenic (35, 37). Potential increased fungal tissue adherence permits invasion of the already compromised or even repairing tissue containing high levels of fibrin/fibrinogen (35, 37, 45). Extensive epithelial damage could expose receptors/sensors (melanin-sensing C-type lectin receptor (MelLec)) to *Aspergillus* found on endothelial cells and promote infection, particularly if the mechanism is already compromised by host genetic variation (45). Risk is possibly enhanced by immune dysregulation, such as lymphopenia which is already a risk factor for IA and possibly CAM, but is also a risk factor for PcP, not a common complication in COVID-19 (25, 37, 47). Hypoxia, common in severe COVID-19 cases, possibly modulates host immune response to fungi and has been shown to limit cytokine release from and maturation of dendritic cells stimulated with *A. fumigatus* (48). Increased platelet counts, common in COVID-19 patients with severe disease, associated with vascular complications may increase risk of co-infections (49, 50).

Underlying clinical conditions (e.g. solid organ transplantation, solid cancer, chronic respiratory conditions, diabetes) that predispose to IFD, have also been associated with risk of COVID-19 associated IFD, particularly when management of that condition already involved immuno-suppressive therapy, that was continued or potentially enhanced for the management of COVID-19 (26, 51, 52). With corticosteroids dampening the hosts pro-inflammatory response but also immune response and clearance of pathogens, several studies have suggested an association between their administration and increased risk of IFD with 52% of CAPA and 37% (*P*:0.0076) of non-CAPA patients receiving them during their care (25, 26, 52, 53). Systemic corticosteroid use is also common (79%) in patients with CAM and while exhibiting the typical effects on host immunity (impaired neutrophil migraton, ingestion and phagolysosome fusion), it limits the efficacy of insulin increasing the chances of uncontrolled diabetes which itself is a risk factor for CAM (37). Mucorales spp. need free iron for essential metabolic processes (54). In patients with poorly controlled/uncontrolled diabetes, hyperglycaemia and the subsequent ketoacidosis leads to high levels of free iron in the circulation, something potentially enhanced with corticosteroids and raised free iron levels are common in the serum of the COVID-19 patient with or without ketoacidosis (37). Raised circulatory free iron levels may also be associated with renal failure in the COVID-19 patient undergoing deferoxamine chelation, with deferoxamine being a siderophore for Mucorales spp (37).

During COVID-19 infection glucose receptor protein 78 (GRP78) is upregulated, potentially serving as a host factor for viral entry and infection, with GRP78 induced by high concentrations of glucose, iron and ketone bodies, all present in the patient with poorly controlled diabetes (55). During mucormycosis, the spore coat protein homologue CotH3, present on the surface of the conidia, promotes infection *via* its interaction with GRP78 present on host nasal epithelial cells (56). The potential for increased concentrations of GRP78 during COVID-19 infection could increase risk of mucormycosis and being located in the nasal cavity potentially explain the large number of cases of rhino-orbital-(cerebral) mucormycosis.

Regulation of the inflammatory response to COVID-19 may be critical to minimizing susceptibility to secondary fungal infection, but must be balanced against maintaining sufficient natural host immunity against fungal pathogens. In addition to corticosteroid use, immunomodulatory therapies inhibiting IL6 (tocilizumab/sarilumab) and Janus kinase (JAK) are recommended for managing COVID-19, with the aim of reducing the cytokine storm or inhibiting signaling required for immune activation (57). *Via* signal transducer and activator of transcription 3 (STAT3), IL6 is critical for protective Th17 anti-*Aspergillus* host responses and given the role these systems have in the immune response to opportunistic infections, including IFD, immuno-modulation will likely increase the risk of IFD, as has been seen in other cohorts at risk of IFD when treated with immunomodulatory therapies (5, 30). In one of the largest, multicentre studies to date, the use of anti-IL6 treatments was associated with increased of IFD in the COVID-19 cohort (OR: 2.93, *P*: 0.0017), and the dual use of dexamethasone and anti-IL6 treatments were significantly associated with CAPA (OR: 2.7, *P*:0.027) (51). Other large studies have confirmed the individual use of dexamethasone and antiIL6 treatment as independent risk factors for CAPA. Although a recent study found similar levels of IL6 (and Pentraxin-3) in the BAL fluid of COVID-19 patients irrespective of CAPA (23, 58, 59). Levels of epidermal growth factor, platelet derived growth factor and CXCL2 were elevated in BAL fluid from CAPA patients (23). Further research into patients with COVID-19 patients admitted to the ICU indicated that there was no significant link between the use of corticosteroids for treatment of COVID-19 and the development of IFD, but more so the prolonged use of corticosteroids to treat pre-existing conditions (60).

Predisposition to CAPA may be related to individual host genetics. In patients, particularly males with CAPA, levels of IL-8 and caspase-3 can be significantly lower, both of which are usually elevated in patients with IA, COVID-19 and the inability to muster a sufficient response may indicate a risk for developing IA during COVID-19 infection (61). Although higher concentrations of IL-8 have been documented in CAPA patients (23). The expression of 34 genes have been shown to be downregulated during CAPA compared to COVID-19 alone, 28 of which were also downregulated during IAPA, and were mainly associated with type I/type II IFN and TNF signaling, and neutrophil degranulation (23). A previously mentioned feature of COVID-19 is the production of autoantibodies against IFNs; this can reduce the effectiveness of these cytokines and related immune functions making these autoantibodies a risk factor for IFD during COVID-19 (24). Gene expression analysis from peripheral blood mononuclear cells confirmed these as risk factors for developing CAPA.

While the increased rates of IC during COVID-19, immunomodulatory interventions have been proposed as a possible risk factor for CAC but initial studies documenting the association lacked significant control cohorts (62). However, a recent study involving 215 COVID-19 patents with an incidence of CAC of 14.4% demonstrated a significant association between potential CAC and the use of tocilizumab with or without corticosteroids (63). In another study, both the use of corticosteroids or immunomodulatory therapy was significantly greater in CAC patients compared to patients with candidaemia in the absence of COVID-19 (34). Given the significant number of other clinical interventions associated with increased risk of IC and widely applied in the ICU, it can be hypothesized that immunomodulatory therapies add to the cumulative clinical risk encountered in critical care (34). The situation with CAC is complicated, the multitude of factors affecting the at-risk population can make it difficult to discern specific effects, a retrospective study of 264 patients did not find an increased risk of IC in patients with severe COVID-19 related pneumonia (64). There is a requirement for further research into the occurrence and risk factors for CAC (65).

### 2.1 Immune exhaustion

The development of secondary infections is usually attributed to direct damage to epithelial membranes or because of drugs such as antibiotics whose activity can lead to dysbiosis and opportunities for infection such as oral candidiasis (39). The risk of IFD is greatly increased in neutropenic patients or those being treated with corticosteroids, as previously discussed. Another mechanism that can increase the risk of infection is immune exhaustion (66). This phenotype in immune cells is where the cells do not perform their expected function by failing to produce cytokines or identify pathogens, it may also be considered expression of inhibitory markers (66).

The major groups of cells that have been studied regarding immune exhaustion are T cells, usually CD8 cytotoxic T cells in viral infections, and NK cells (67, 68). In cases of viral infection immune exhaustion is associated with prolonged infection and inability to clear the pathogen. It is possible to monitor the exhaustion of T cells by measuring programmed death 1 (PD-1), which has been found to be upregulated by exhausted T cells (67). In hepatitis C virus (HCV) infection, the induction of the inhibition receptor NKG2A is an indicator of NK cell exhaustion, these exhausted cells show decreased IFN-γ (68). In both cases blocking PD-1 and NKG2A led to restored antiviral activity. The function of NK cells has been shown to be impaired during COVID-19 infection, possibly increasing risk of IFD and other co-infections (69). NK cell exhaustion has also been observed in an *in vitro* interaction with *A. fumigatus* germlings (70); during this interaction there was a contact-dependent reduction in the expression of the activating receptor NKG2D and IFN-γ.

The role of NK-cells in antifungal immunity has become increasingly clear over the last decade, the role of NK cell derived IFN-γ was essential during the early stages of infection in a murine model (71, 72). NK cells that are in the process of recovering after allogeneic stem cell transplantation displayed an impaired response against *A. fumigatus* indicating an increased host susceptibility to fungal infection (73). Further studies found cytotoxic effect of NK cells against *A. fumigatus in vitro*, leading to the proposal of using NK cells in antifungal therapy (74, 75). There is evidence that antifungal activity is morphotype-type dependent with greatest fungicidal activity against conidia (75).

T cells in COVID-19 patients often show a hyperactive and exhausted phenotype with coinhibitory molecule expression (e.g., PD-1, T cell immunoreceptor with Ig and ITIM domains (TIGIT), and Cytotoxic T-lymphocyte associated protein 4 (CTLA-4)) and increased populations of potentially pathogenic Th17 cells. These attenuated yet sustained adaptive immune responses may be explained by potential activation of bystander T cells that cause organ damage and epitope spreading. Delayed IFN-γ or FS-7-associated surface antigen (Fas)/Fas ligand signaling may trigger apoptosis of virus-specific T cells in activation-induced cell death, as suggested in SARS-CoV-2–infected spleens. Other potential mechanisms for lymphopenia include the direct viral infection of lymphocytes or suppression from other immune cells, perhaps through interactions with tolerogenic mixed M2 myeloid populations (76).

Immune exhaustion directly affects subsets of immune cells needed to effectively control fungal infection. This has been recently shown in a work by Vu et al. (77), who demonstrated in a murine model that the blockade of PD-1 on post-sepsis aspergillosis reinvigorated exhausted antigen-presenting cells and T cells by upregulating CD86 expression and IFN-γ production, and dampened IL-10 production. In consequence, in their model this led to the attenuation of secondary aspergillosis. The authors concluded that adjunctive anti-PD-1 therapy may become a promising strategy for advanced immunotherapy against lethal fungal infection. Corresponding results were also shown for pulmonary infections with Mucorales (78), here the authors demonstrated that inhibition of the PD-1/PD-L1 pathway effectively counteracted T-cell exhaustion, elicited strong pulmonary release of proinflammatory cytokines and chemokines, and revealed improved clinical outcomes of invasive pulmonary mucormycosis in immunosuppressed mice, even without concomitant antifungals.

Severe viral infection often causes a septic cytokine storm followed by immune exhaustion/paralysis. Not surprisingly, many pathogens are equipped with various anti-inflammatory mechanisms. Such mechanisms might be leveraged clinically to control septic cytokine storms. As an example, the N-glycan from *C. albicans* ameliorates mouse sepsis through immunosuppressive cytokine IL-10. In a sepsis model using lipopolysaccharide (LPS), injection of the N-glycan upregulated serum IL-10, and suppressed pro-inflammatory IL-1β, TNF and IFN-γ. The N-glycan also improved the survival of mice challenged by LPS. Analyses of structurally defined N-glycans from several yeast strains revealed that the mannose core is key to the upregulation of IL-10. Knocking out the C-type lectin Dectin-2 abrogated the N-glycan-mediated IL-10 augmentation. Furthermore, *C. albicans* N-glycan ameliorated immune exhaustion/immune paralysis after acute inflammation (79).

Not only in SARS-CoV-2 infection, both, the reduced numbers, and cytotoxic activity of circulating NK, CD4+ and CD8+T cells contribute to suboptimal outcomes, this phenomenon has also been seen in H1N1 influenza, Epstein Barr Virus (EBV), SARS-COV and Middle East respiratory syndrome (MERS) infections, although not as frequently (80).

### 2.2 COVID-19 induced immune exhaustion and IFD

There is now evidence that immune exhaustion is a component of the immune dysregulation caused by SARS-CoV-2 infection (81). COVID-19 patients display reduced numbers of CD8 T cells and NK cells, these also show increased PD-1 and NKG2A which, as previously discussed are indicators of immune exhaustion (81, 82). This immune exhaustion is connected to the prolonged immune response induced during COVID-19 (83). Further studies have measured decreased IFN-γ and TNF production by NK cells as seen in other viral infections (84), creating risk factors for IFD as observed in other infections.

The potential for COVID-19 induced immune exhaustion to increase the risk of IFD has so far been the focus of very few studies. The observed reduction in T and NK cells in COVID-19 and the presence of exhaustion markers have been proposed as a contributor to the incidence of CAM (85). An analysis of patient characteristics in India also found that indicators of T cell exhaustion and prolonged periods with reduced T cell numbers were important for the development of CAM in diabetics (86).

An example of this COVID-19 associated immune exhaustion was demonstrated using gene expression profiling of BAL samples from COVID-19 patients with and without aspergillosis (23). The patients with CAPA had downregulated type II IFN signaling compared with patients not affected by aspergillosis. This suppression of the pro-inflammatory antifungal host response might have severe consequences on the activation of many immune cell populations for instance *via* inducing microtubule-associated protein 1A/1B-light chain 3 (LC3)-associated phagocytosis.

A functional analysis to determine the connection between immune cells from COVID-19 patients and fungal pathogens demonstrated an impaired immune response to fungal infection (87). Challenging T cells from COVID-19 patients with antigens from *A. fumigatus* and *Rhizopus arrhizus* revealed impaired cytokine expression and increased expression of the exhaustion marker PD-1 (87). A significant finding in this study was impaired fungicidal activity by neutrophils, neutropenia is a significant risk factor for IFD and neutrophils are the most important innate immune cells in antifungal immunity (88). Neutrophils will typically kill conidia after phagocytosis, impaired killing could lead to fungal persistence within neutrophils or the uncontrolled growth of germlings leading to invasive infection (89). SARS-CoV-2 infection disrupts the innate and adaptive immune systems in a manner that promotes the development of IFD independent of medical intervention.

## 3 Concluding comments

**Figure 1:**
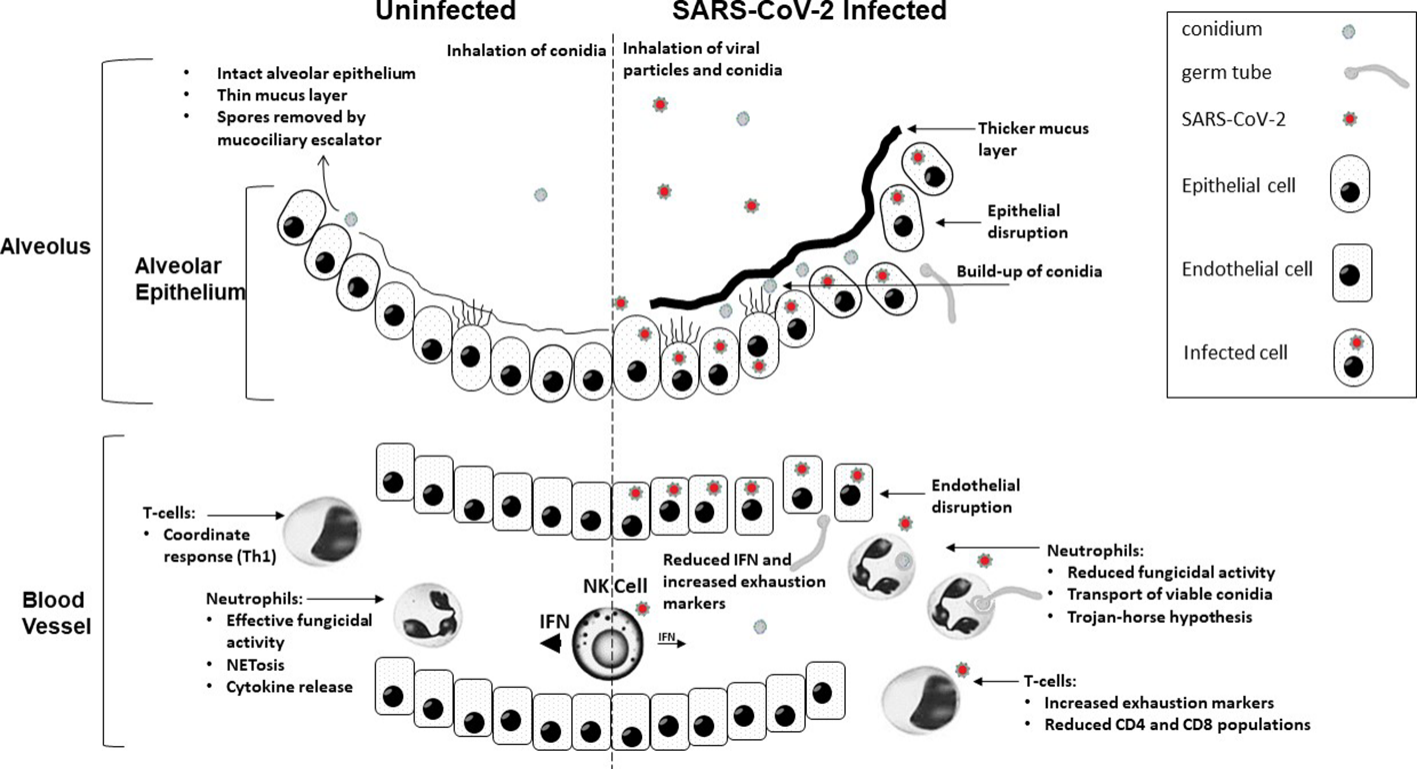
A graphical summary demonstrating how COVID-19 infection contributes to increased susceptibility to invasive fungal disease.

During COVID-19 infection immune dysregulation increasing the risk of secondary IFD is likely a combination of clinical intervention/immunosuppression, host factors (existing underlying conditions and/or host predisposition) and the pathophysiology of COVID-19 infection. A three-phase breakdown in the innate antifungal immunity has been proposed, where, initially, severe disruption to the respiratory epithelium resulting from an excessive pro-inflammatory response to COVID-19 infection and limited mucociliary clearance provides ample opportunity for fungal tissue invasion. This is compounded by impaired phagocytosis of conidia, a result of down-regulated expression of genes associated with phagocytic opsonisation, recognition and conidial killing and once conidia have evaded this second phase, hyphal growth is not controlled due to lower neutrophil fractions in the third phase (23). However, immune exhaustion likely plays a role and is well documented during COVID-19 infection, and host immunity, post even moderate disease, has been shown not to respond as expected to the presence of fungi. Given the complexity of this picture, a range and combination of factors will likely predispose individual COVID-19 patients to IFD, and a single predisposing profile is highly unlikely (**Table 1** and **Figure 1**). Understanding the individual immune status will likely govern the degree of tissue and angio-invasion, and will be critical to optimizing patient specific immunotherapy when managing cases of respiratory virus associated fungal disease. Gaining an understanding of which patient would benefit from suppressing hyper-inflammatory damage through anti-inflammatories versus the use of adjuvant stimulatory immunotherapy (recombinant IFN-γ) will be dependent on improving our understanding of respiratory virus pathogenicity and its subsequent influence on the risk of IFD (23, 93). Through improved understanding of the various host immune and clinical risk factors it may be possible to develop predictive algorithms that can be coupled with existing diagnostics to identify high risk patients and possibly apply pre-emptive strategies, provide early diagnosis and treatment, or a prognostic indication during the COVID-19 pandemic, but more so prepare us for future issues.

**Table 1:** Summary of the effects of SARS-CoV-2 infection on the host that match risk factors for the development of IFD.

Effects of COVID-19 on Host	Risk Factors for IFD
COVID-19 binds to ACE-2 receptors on respiratory epithelial cells Poorly regulated pro-inflammatory response (see below) leading to impaired acquired immunity and dysregulated innate response. Direct viral infection of lymphocytes (71) Reduction of Dendritic cells, their maturation and function (41) T cell effects: Reduced CD4+ and CD8+ populations (82) *PD-1 upregulation (67)	Limited muco-ciliary clearance and innate function (37) Cytokine storm and extensive damage to lung epithelium. Lymphopenia Lymphopenia Limited activation of T-cells Dysregulation of Th1 responses and cytokine signaling increases risk of IFD (80, 87) Marker of immune exhaustion (62), limited T-cell response to Mucorales and *Aspergillus* antigens (82)
NK Cells: *NKG2A upregulation (68, 70) Reduced IFN-γ release (87)	Functional NK cells (71) and NK cell derived IFN-γ necessary for antifungal immunity (72)
Monocytes/Neutrophils: Reduced fungicidal activity (87) Redistribution of monocyte population/immature neutrophils	Neutropenia and neutrophil defects are important for IFD (88) Hyper-inflammatory response (42) leading to increased pulmonary fibrosis (43)
Abnormal lung function and epithelial damage (90)	Decreased removal of spores and epithelial damage increase the risk of IFD (37). Potential for increased fungal adherence to fibrin/fibrinogen in damaged tissue. Exposure to endothelial receptors associated with infection (43). Hypoxia impacts cytokine release and maturation of dendritic cells (46).
Corticosteroid treatment (25, 26) IL6 inhibitors (55) Increased concentrations of GRP78	Immunosuppressive treatment is a key risk factor for IFD (6) Via STAT3, IL6 response is critical to a TH-17 anti-*Aspergillus* response (29) Promotes infection with Mucorales spp. *via* CotH3 (54)
Genetic polymorphisms affect COVID-19 infection (91). Link between COVID-19 polymorphisms and IFD not yet known. Altered Gene expression (genetic predisposition to IFD)	Genetic polymorphisms in genes such as CARD9 increase the risk of IFD (92) 34 genes, were mainly associated with type I/type II IFN and TNF-α signaling, and neutrophil degranulation were downregulated in CAPA pts (23). IL8 and Caspase 3 significantly lower in CAPA pts (58)

*Markers of cellular exhaustion.

ACE-2, Angiotensin-converting enzyme 2.

Th-1, Type 1 helper (Th-17, Type 17 helper).

CD4, Cluster of differentiation 4.

CD8, Cluster of differentiation 8.

IFD, Invasive fungal disease.

PD-1, Programmed cell death protein 1.

NK, Natural killer cells (NKG2A, Natural killer cell inhibition receptor G2A).

IFN, Interferon.

IL, Interleukin.

STAT-3, Signal transducer and activator of transcription 3.

CotH3, spore coat protein homolog 3.

CARD9, Caspase recruitment domain-containing protein 9.

TNF, Tumor Necrosis factor.

CAPA, COVID-19 associated pulmonary aspergillosis.

## Author contributions

CM and PLW: Conceptualization. All authors: Writing and proof-reading. All authors contributed to the article and approved the submitted version.

## Conflict of interest

The authors declare that the research was conducted in the absence of any commercial or financial relationships that could be construed as a potential conflict of interest.

The handling editor SW declared a past collaboration with the authors CM, JL, LW.

## Publisher’s note

All claims expressed in this article are solely those of the authors and do not necessarily represent those of their affiliated organizations, or those of the publisher, the editors and the reviewers. Any product that may be evaluated in this article, or claim that may be made by its manufacturer, is not guaranteed or endorsed by the publisher.
